# From Circulation to Regeneration: Blood Cell Membrane-Coated Nanoparticles as Drug Delivery Platform for Immune-Regenerative Therapy

**DOI:** 10.3390/pharmaceutics18010066

**Published:** 2026-01-04

**Authors:** Yun-A Kim, Min Hee Lee, Hee Su Sohn, Han Young Kim

**Affiliations:** 1 Department of Biomedical-Chemical Engineering, The Catholic University of Korea, Bucheon 14662, Republic of Korea; 2 Department of Biotechnology, The Catholic University of Korea, Bucheon 14662, Republic of Korea; 3 Division of Pulmonary and Critical Care Medicine, Department of Medicine, Brigham and Women’s Hospital, Harvard Medical School, Boston, MA 02115, USA; 4 Research Institute for Controlled Biomaterials of Regulated Cell Death, The Catholic University of Korea, Bucheon 14662, Republic of Korea

**Keywords:** cell membrane-coated nanoparticles, blood cell membranes, hybrid biomimetic systems, drug delivery, regenerative medicine, translational nanomedicine

## Abstract

Cell membrane-coated nanoparticles represent a biomimetic drug delivery approach that integrates biological membrane functions with synthetic nanomaterials. Among the various membrane sources, those derived from blood cells such as red blood cells, platelets, and leukocytes offer distinctive advantages, including immune evasion, prolonged systemic circulation, and selective tissue targeting. These properties collectively enable efficient and biocompatible delivery of therapeutic agents to diseased tissues, minimizing off-target effects and systemic toxicity. This review focuses on blood cell membrane-derived nanocarriers as drug delivery and immune-regenerative platforms, in which membrane-mediated immunomodulation synergizes with therapeutic payloads to address inflammatory or degenerative pathology. We discuss recent advances in blood cell membrane coating technologies, including membrane isolation, nanoparticle core selection, fabrication techniques, and the development of hybrid and engineered membrane systems that enhance therapeutic efficacy through integrated immune regulation and localized drug action. To illustrate these advances, we also compile membrane type-specific nanocarrier systems, summarizing their core nanoparticle designs, coating strategies, therapeutic cargoes, and associated disease models. Challenges related to biological source variability, scalability, safety, and regulatory standardization remain important considerations for clinical translation. In this review we systematically address these issues and discuss emerging solutions and design strategies aimed at advancing blood cell membrane-based nanocarriers toward clinically viable immune-regenerative therapies.

## 1. Introduction

Over the past decade, cell membrane-coated nanoparticles have emerged as a crucial biomimetic platform for enhancing drug delivery systems [1,2]. By covering synthetic nanoparticle cores with natural cell membranes, researchers have created carriers that preserve the key surface proteins and biological properties of their source cells [3,4]. Among the various cell types available, blood cells have received particular attention because of their abundance and unique biological roles [5]. Red blood cells (RBCs), platelets, and white blood cells (WBCs) also known as leukocytes, have each been used to develop biomimetic nanoparticles that take advantage of their native biological characteristics [6,7]. A key motivation for using blood cell membranes is to overcome the rapid clearance and immune recognition that often limit the performance of unmodified nanoparticles [8]. Bare nanoparticles are quickly opsonized by plasma proteins and removed by the mononuclear phagocyte system, reducing their therapeutic efficiency [9]. In contrast, coating nanoparticles with self-derived membranes disguises them as endogenous, decreasing phagocytic uptake [4]. For instance, the RBC membrane protein CD47 interacts with macrophage receptors to send a “don’t eat me” signal [10]. Platelet membranes contain complementary regulatory proteins such as CD55 and CD59, which help suppress immune activation [11]. Together, these properties help nanoparticles remain in circulation for longer periods and improve drug bioavailability [12]. Blood cell membranes also provide natural targeting abilities [13]. Neutrophil membrane nanoparticles exhibit CXCR2- and β2-integrin-mediated inflammatory homing, enabling active recruitment into inflamed endothelium [14]. When used as coatings, these membranes transfer their natural targeting traits to the nanoparticles, enabling site-specific drug delivery without synthetic ligands [15]. For example, neutrophil membrane-coated nanoparticles can migrate to inflamed areas [7], while platelet-coated nanoparticles can accumulate in tumor microenvironments by binding to endothelial receptors [16]. Accordingly, blood cell membrane-coated nanoparticles have been explored across a range of therapeutic fields including cancer therapy, inflammation control, and infection treatment [17]. These systems improve the targeted delivery of chemotherapeutics, photosensitizers, and immune modulators, thereby increasing therapeutic efficacy while reducing side effects [18].

Beyond immune evasion or tissue targetability, blood cell membranes retain intrinsic immunoregulatory programs that allow them to sense inflammatory cues and participate in immune homeostasis. Blood cell membranes preserve a broad spectrum of native surface ligands, receptors, and regulatory proteins that directly engage immune cells and inflammatory microenvironments, enabling membrane-driven immune modulation independent of loaded therapeutics [11]. Through preserved membrane proteins involved in cell–cell recognition and immune signaling, these membranes can partially regulate immune activation, leukocyte recruitment, and inflammatory signaling at diseased sites [19,20,21]. Such immune modulation is increasingly understood to initiate regenerative cascades by creating a pro-regenerative microenvironment, characterized by inflammation resolution, permissive angiogenic signaling, and enhanced survival and recruitment of reparative cells [19,20,21]. However, membrane-mediated immune modulation alone is often insufficient to fully resolve established inflammatory or degenerative pathology, revealing a critical limitation of membrane-only systems [22]. Accordingly, the integration of therapeutic agents into blood cell membrane-coated nanoparticles should be understood as a synergistic extension of membrane-driven immune regulation rather than a parallel strategy. The membrane component enhances circulation stability, immune compatibility, and tissue-specific accumulation, while the therapeutic cargo amplifies and stabilizes immune-regenerative signaling within the targeted microenvironment. This cooperative interaction enables blood cell membrane-coated nanoparticles to function as unified immune-regenerative therapeutic platforms, in which membrane-mediated biological recognition and drug-mediated intervention operate as a single integrated mechanism [19]. Membrane-centric immune interaction distinguishes blood cell membrane-coated nanoparticles from naturally secreted extracellular vesicles (EVs) such as exosomes. Although exosomes possess intrinsic bioactivity, their limited production yield and small luminal volume constrain scalable manufacturing and drug loading capacity [23], thereby restricting their efficiency as drug delivery platforms. In contrast, blood cell membrane-based systems offer higher material availability, improved scalability, and greater flexibility in cargo incorporation, while maintaining immune-interactive properties essential for targeted and regenerative therapy [17].

**Figure 1:**
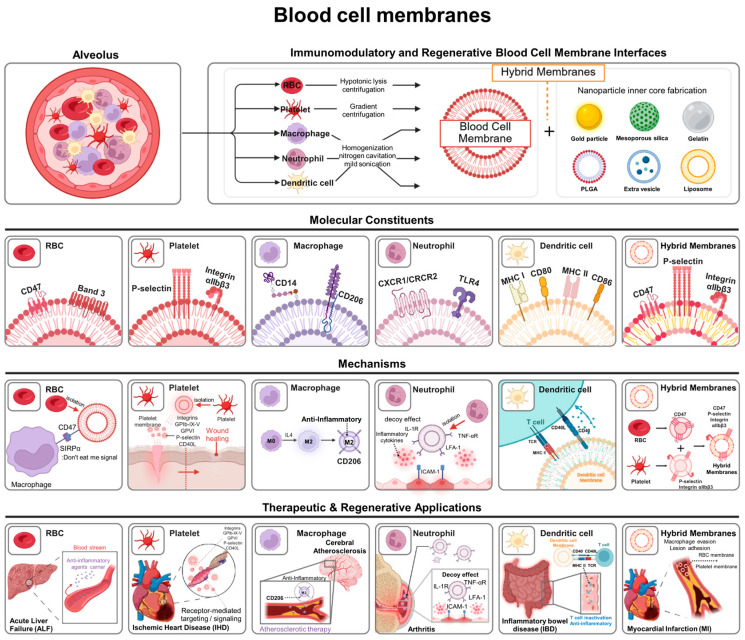
Overview of blood cell membrane-coated nanoparticles: mechanisms and applications in regenerative therapeutics.

In this review, we frame blood cell membrane-coated nanoparticles as immune-regenerative therapeutic platforms, emphasizing their role as active biological interfaces that reshape immune recognition and inflammatory signaling to promote tissue repair, rather than as passive carriers designed solely for drug transport [5]. To provide an overview of these mechanisms and therapeutic applications, the major biological features and regenerative interactions of blood cell membrane-coated nanoparticles are summarized in Figure 1. This review focuses on the therapeutic rather than diagnostic use of blood cell membrane-coated nanoparticles. The following sections first describe fabrication and membrane-coating strategies for blood cell membrane-based nanocarriers, followed by a systematic overview of blood cell membrane sources and their roles in drug delivery and regenerative applications. We highlight how membrane-mediated immune interactions facilitate targeted drug delivery and cooperate with therapeutic payloads to modulate immune responses that ultimately support regenerative outcomes. Subsequent sections address key translational challenges and conclude with future perspectives. Together, this review provides a comprehensive and integrated perspective on blood cell membrane-based nanocarriers, spanning their design principles, biological functions, therapeutic applications, and translational considerations within the broader context of immune-regenerative drug delivery.

## 2. Fabrication of Blood Cell Membrane-Integrated Nanocarriers

### 2.1. Cell Membrane Isolation

**Figure 2:**
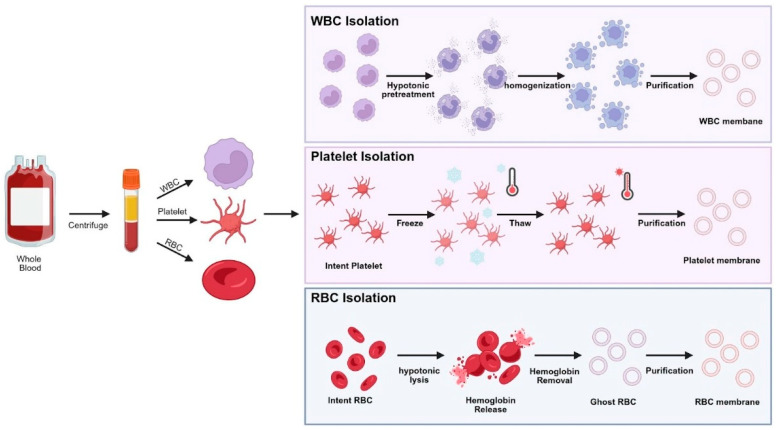
Workflow for cell type-specific membrane extraction from whole blood.

The preparation of blood cell membrane-coated nanoparticles begins with the careful isolation of intact cell membranes. Cell membrane coating is typically achieved through physical membrane–nanoparticle fusion using extrusion, sonication, or microfluidic approaches (Figure 2). These membranes are easily collected in large quantities and can be purified through simple centrifugation steps. Among the various cell types, RBCs and platelets are the most widely used because they are anucleate [1,5,7]. RBC membranes are typically obtained through hypotonic lysis, which removes intracellular contents while leaving the plasma membrane intact and flexible [3,7]. Platelet membranes are usually isolated by sequential washing and density-gradient centrifugation, preserving key surface proteins such as CD47, P-selectin, and CD41 that are essential for immune evasion and biological activity [7,11].

For nucleated cells such as leukocytes including neutrophils or macrophages, more delicate disruption methods are necessary. Techniques including mild sonication, mechanical homogenization, or nitrogen cavitation are used to rupture the plasma membrane while minimizing contamination from intracellular organelles [13,15]. After disruption, the suspension is purified by differential centrifugation and density-gradient separation to obtain a clean plasma membrane fraction. Additional steps are applied to remove residual contaminants, such as DNase treatment to eliminate nucleic acids, high-salt washes to remove plasma proteins, and endotoxin-removal resins to ensure safety [5]. Filtration through a membrane pore helps remove debris, though care must be taken not to damage the delicate bilayer structure.

Membrane integrity and purity are evaluated by several analytical techniques. Protein content and identity are confirmed using SDS-PAGE and Western blotting for specific markers, while lipid composition can be analyzed through lipidomics [4]. Physical characteristics such as size and charge are assessed using dynamic light scattering and zeta potential analysis. Morphological features and bilayer uniformity are often examined using transmission electron microscopy or atomic force microscopy. The correct orientation of transmembrane proteins, which is essential for biological function, can be verified through flow cytometry or immunogold labeling assays.

### 2.2. Core Platforms for Membrane Integration

The choice of nanoparticle core plays a crucial role in determining the performance of the final membrane-coated system. Different materials influence drug loading, release kinetics, biocompatibility, and overall therapeutic behavior [1,24]. Biodegradable polymers such as polylactic-co-glycolic acid (PLGA) and polylactic acid (PLA) are among the most common choices because their size, surface charge, and degradation rate can be finely tuned [25]. Protein-based materials such as albumin nanoparticles offer excellent biocompatibility and a wide range of surface modification possibilities [26]. Lipid-based carriers, including liposomes and lipid nanoparticles (LNPs), exhibit high fusogenicity, which allows them to blend naturally with biological membranes [15]. Inorganic nanoparticles such as gold, silica, and iron oxide are also used, particularly when additional imaging, photothermal, or magnetic properties are desired [27,28]. Recently, hybrid organic-inorganic nanoparticles have gained attention for their ability to combine the structural stability of inorganic materials with the functional versatility of organic polymers [17,29].

Beyond conventional coating, lipid-based carriers can spontaneously fuse or intercalate with biological membranes to form hybrid bilayers [30]. This approach has been successfully applied to RBC membrane–liposome and platelet membrane–liposome systems, where partial lipid mixing enhances colloidal stability and preserves membrane protein orientation [31,32]. Moreover, hybrid organic–inorganic platforms now integrate these fusogenic features to achieve synergistic biointerfacing and prolonged circulation [33].

The surface properties of the nanoparticle core strongly affect the efficiency of membrane coating. A slightly negative or near-neutral zeta potential tends to favor stable membrane adhesion, while excessive roughness or high hydrophobicity can hinder bilayer fusion [4,34]. For this reason, many nanoparticles are pretreated with PEGylation or amphiphilic linkers to improve membrane fusion and ensure uniform coverage [34]. These surface treatments also help maintain colloidal stability and prevent aggregation during storage or in biological fluids [4].

### 2.3. Membrane Fusion and Coating Techniques

A variety of fabrication techniques have been developed to combine purified cell membranes with nanoparticle cores. Each method aims to achieve a uniform, right-side-out coating while maintaining the structural and functional integrity of membrane proteins. The most established approach is mechanical extrusion, in which a mixture of membrane vesicles and nanoparticles is repeatedly passed through polycarbonate membranes with defined pore sizes. This method produces uniform particles and helps align the membrane in the correct orientation [35]. However, it is a batch process and can be difficult to scale up. Another widely used method is sonication, where ultrasonic energy drives the fusion of membranes onto nanoparticle surfaces. Although effective, it requires careful optimization because excessive power or duration can denature sensitive membrane proteins. Co-incubation is a simpler, passive technique that relies on natural electrostatic and hydrophobic interactions between membranes and nanoparticle cores. While it is easy to perform, it often produces heterogeneous coating thicknesses [4]. More recently, microfluidic electroporation has emerged as a promising strategy for large-scale and highly reproducible membrane coating [33,36]. Beyond coating, membrane fusion or hybridization strategies have attracted increasing attention, with systems such as RBC membrane–LNP and platelet membrane–EV hybrids showing enhanced fusogenicity and biological compatibility [33]. Similarly, RBC–platelet hybrid membranes demonstrate synergistic immune and regenerative properties. These fused bilayers provide improved mechanical stability, membrane fluidity, and biological interface continuity compared to discrete coatings [37].

Verification of membrane orientation and completeness is essential. Advanced analytical tools such as fluorescence quenching assays, confocal microscopy, and proteomic profiling are frequently used to assess full membrane coverage and preservation of key proteins. As manufacturing technologies evolve, the integration of automated microfluidic systems and closed-loop production platforms is expected to enable large-scale, reproducible fabrication of membrane-coated nanoparticles with clinical-grade quality and consistency [5,36]. At the fabrication level, two distinct membrane-integration paradigms are employed: (i) coating preformed nanoparticle cores with blood cell ghost membranes and (ii) directly converting ghost membranes into nanoscale vesicles without a synthetic core. In the former approach, additional purification is required to selectively isolate fully coated nanoparticles from uncoated cores and membrane-only vesicles, which is commonly achieved using density-gradient centrifugation, size-exclusion chromatography, or affinity-based separation methods. Quantitative validation of membrane orientation is essential to confirm correct right-side-out presentation and is typically performed using protease protection assays, fluorescence quenching, or antibody accessibility measurements combined with flow cytometry or fluorescence spectroscopy [38].

## 3. Blood Cell Membrane Sources: Drug Delivery Uses and Regenerative Applications

### 3.1. RBC Membranes

RBCs are one of the most widely used membrane sources for nanoparticle coatings because they are abundant, easily isolated, and highly biocompatible. Their membranes possess unique mechanical and biochemical features that provide both stability and immune evasion. The spectrin–actin cytoskeleton, anchored by ankyrin and band-3 complexes, gives RBCs remarkable elasticity and mechanical strength, allowing them to pass through narrow capillaries without rupturing [32,39]. The RBC lipid bilayer displays distinct asymmetry, with phosphatidylserine and phosphatidylethanolamine concentrated on the inner leaflet, and phosphatidylcholine and sphingomyelin enriched on the outer side [30]. This organization maintains membrane fluidity and prevents unwanted cell aggregation, while negatively charged sialylated glycoproteins reduce nonspecific protein adsorption and complement activation [40]. Key surface proteins such as CD47, CD55, and CD59 protect the cells from phagocytosis and complement-mediated lysis, and the absence of nuclei and mitochondria minimizes metabolic activity and immunogenicity [10]. When used as nanoparticle coatings, RBC membranes significantly extend circulation time and reduce clearance by the mononuclear phagocyte system [3,12].

**Figure 3:**
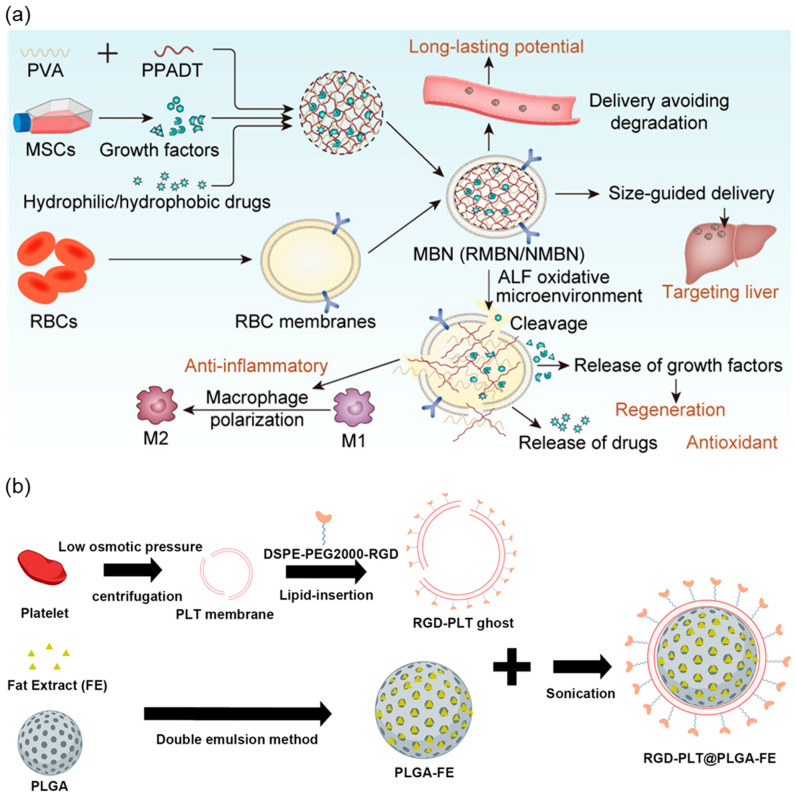
(**a**) RBC membrane-coated nanoframework enhances circulation, reduces inflammation and oxidative stress, and promotes liver regeneration in acute liver failure. Reprinted with permission from Ref. [42] under the term CC-BY license. 2024 ACS Publication. (**b**) Schematic illustration of RGD-modified platelet-membrane-cloaked PLGA nanocarriers for targeted fat extract delivery to ischemic brain vasculature, promoting angiogenesis and neurogenesis. Reprinted with permission from Ref. [58] under the term CC-BY license. 2022 Springer Nature.

Based on these intrinsic properties, RBC membrane-coated nanoparticles have been widely explored as immune-modulatory drug delivery platforms capable of supporting regenerative therapies [31]. Their natural negative surface charge and CD47-mediated immune evasion enable them to avoid clearance by hepatic Kupffer cells, thereby prolonging systemic circulation and enhancing local drug accumulation [10]. Importantly, prolonged circulation does not imply reduced hepatic accumulation in disease models. Instead, extended blood retention increases the likelihood of nanoparticle extravasation into injured liver tissue, where vascular permeability, inflammatory chemotaxis, and microenvironmental disruption facilitate preferential accumulation [41]. As a result, RBC membrane coatings have been shown to improve the retention of anti-inflammatory agents and promote hepatic tissue regeneration (Figure 3a) [42]. In this study, RBC membrane-coated multifunctional nanoframework was constructed, providing antioxidative and anti-inflammatory effects, modulating Kupffer cell activation, and promoting hepatocyte proliferation and tissue restoration in an acute liver failure model. Furthermore, recent study developed mesenchymal stem cell/RBC-inspired nanoparticle that leveraged RBC membranes to protect MSC-derived paracrine factors and showed reduced hepatocellular apoptosis, lower inflammatory cytokine levels, and enhanced hepatocyte proliferation [43]. This hybrid design prolonged circulation time and improved survival outcomes in CCl_4_-induced acute liver injury. Collectively, these findings support that RBC membranes not only act as immune-evasive coatings but also actively participate in the creation of a regenerative hepatic microenvironment [31]. Beyond hepatic injury models, RBC membranes have also demonstrated therapeutic potential in non-cancer regenerative and anti-inflammatory applications. Their prolonged circulation, immune-evasive properties, and intrinsic anti-oxidative activities contribute to tissue protection and repair of ischemic brain [44]. These membrane-based systems have been shown to reduce inflammatory cytokines, attenuate oxidative stress, and promote functional recovery across diverse injury environments, further supporting the concept that RBC membranes can serve as active therapeutic interfaces rather than passive coating materials.

Several intrinsic immunoregulatory and metabolic features of RBC membranes are believed to contribute directly to tissue repair and regeneration under diverse pathological conditions. By regulating nitric oxide metabolism, RBC membranes can enhance microvascular circulation and oxygen delivery in injured tissues, supporting cell survival and promoting structural remodeling [32]. Their interactions with monocytes and macrophages also help shift macrophage polarization toward an anti-inflammatory M2 phenotype, accelerating the resolution of inflammation and facilitating extracellular matrix reconstruction [45,46,47]. The exposure of phosphatidylserine on the membrane surface further recruits regulatory immune cells and maintains an immune-tolerant environment, preventing secondary immune-mediated injury and preserving long-term tissue homeostasis [48,49,50]. Activation of the heme oxygenase-1 pathway during RBC membrane turnover produces cytoprotective molecules such as carbon monoxide and bilirubin, which alleviate oxidative stress while promoting angiogenesis and cellular recovery [51,52]. In addition, membrane-associated proteins and lipid mediators contribute to endothelial function and vascular remodeling, assisting in the restoration of damaged or ischemic microenvironments [53,54]. Collectively, these insights highlight that RBC membranes are not merely passive immune-evasive coatings but bioactive regenerative interfaces capable of orchestrating immune modulation, vascular repair, and tissue regeneration.

### 3.2. Platelet Membranes

Platelet membranes possess a rich composition of cholesterol and sphingomyelin, supported by a dynamic cytoskeletal structure that enables rapid shape changes during vascular injury [11,55]. Upon activation, phosphatidylserine exposure provides a procoagulant surface, while integrins, glycoproteins, and P-selectin facilitate adhesion to damaged endothelium [11,55,56]. In addition, sialylated glycans and CD47 help platelets escape immune surveillance [10]. As biomimetic coatings, platelet membranes endow nanoparticles with lesion-specific targeting and prolonged systemic circulation. Notably, platelet membranes inherently carry growth factors such as PDGF, VEGF, and TGF-β, which collectively promote angiogenesis, fibroblast proliferation, and extracellular matrix remodeling [57]. This regenerative profile has motivated the use of platelet membranes in tissue repair applications.

Preclinical studies using platelet membrane-coated nanoparticles have demonstrated that combining lesion-specific adhesion with growth factor or gene delivery can accelerate wound healing and vascular repair. For example, platelet membrane-cloaked nanocarriers loaded with fat extract have shown selective adhesion to ischemic brain vasculature, enhancing angiogenesis and neurogenesis in ischemic stroke models (Figure 3b) [58]. In deep burn wound models, platelet membrane-coated nanoparticles carrying bFGF and VEGFA genes promoted granulation tissue formation, re-epithelialization, and neovascularization, thereby accelerating wound closure and improving tissue quality [59]. In addition, platelet membrane biomimetic nanoparticles delivering TGF-β1 siRNA have been shown to attenuate renal inflammation and fibrosis by targeting injured renal vasculature and modulating pro-fibrotic signaling [60]. Together, these findings indicate that platelet membrane-derived coatings can couple adhesive lesion targeting with local delivery of regenerative cues across diverse injury settings. Although platelet membranes express adhesion-related proteins such as integrins and selectins, which can undergo activation-dependent conformational changes and promote interactions with endothelial cells or extracellular matrix components, this does not necessarily contradict the circulation benefits observed for platelet membrane-coated nanoparticles. In most reported studies, the relevant comparison is between bare nanoparticles and platelet membrane-coated nanoparticles, rather than between free platelets and other blood cell types. In this context, platelet membrane coating provides partial immune camouflage and reduces nonspecific opsonization relative to uncoated particles, resulting in prolonged circulation compared to bare nanoparticles, despite the intrinsic adhesive properties of platelet-derived membranes [10,61,62]. Importantly, platelet membrane-based systems are primarily designed to exploit vascular adhesion and lesion-specific targeting rather than to maximize systemic persistence. Therefore, even when circulation half-life is shorter than that achieved with other blood cell membrane coatings, enhanced local accumulation at injured or inflamed sites can compensate for reduced systemic exposure and contribute to improved therapeutic outcomes [58,60].

The regenerative potential of platelet-related systems also extends to bone and dental tissues. Injectable platelet-rich fibrin (PRF) has been reported to positively regulate osteogenic differentiation of stem cells at implant sites through ERK1/2 signaling, supporting bone–implant integration [63]. Moreover, nanoparticle-based composite coatings that provide controlled release of angiogenic and osteoinductive factors such as VEGF and BMP-2 have been shown to enhance bone regeneration and osseointegration, illustrating how growth factor-releasing nanocarriers can be integrated into hard-tissue repair strategies [64]. Recent work further suggests that incorporating platelet membrane-inspired or platelet-derived components into hydrogel matrices may enhance collagen deposition, angiogenesis, and implant stabilization, positioning platelet-based interfaces as versatile platforms for soft and hard tissue regeneration [61].

Beyond their regenerative capabilities, platelet membrane-coated nanoparticles also exert important immune-modulatory effects during tissue repair. Platelet membrane-coated nanoparticles have been shown to suppress excessive platelet activation and the formation of neutrophil extracellular traps (NETs) in an acute lung injury model, leading to reduced inflammation and improved pulmonary recovery [19]. This work revealed that platelet membranes can directly modulate innate immune pathways while facilitating tissue regeneration. In ischemic stroke models, platelet membrane-coated nanocarriers selectively adhere to damaged vascular endothelium, where they deliver anti-inflammatory payloads and angiogenic factors to restore local blood flow and support neurovascular repair [58,62]. Collectively, these studies indicate that platelet membrane-based nanocarriers integrate adhesive targeting, immune modulation, and intrinsic regenerative signaling, making them a versatile platform for wound healing, vascular repair, and inflammation-associated tissue regeneration.

### 3.3. Leukocyte Membranes

Leukocytes (WBCs), including neutrophils, monocytes or monocyte-derived macrophages, dendritic cells, and natural killer (NK) cells, play central roles in innate and adaptive immune responses and exhibit intrinsic homing behavior toward sites of inflammation, infection, or tissue injury [65]. This innate migratory capability enables leukocyte-derived membrane coatings to function as powerful targeting interfaces for transporting therapeutic nanoparticles into diseased microenvironments [66].

Their plasma membranes retain key surface receptors, including chemokine receptors, integrins, and pattern-recognition molecules, which enable selective targeting of inflammatory tissues and immune modulation, making them highly attractive candidates for biomimetic drug delivery and regenerative applications. In particular, neutrophil- and macrophage-derived membranes have been widely explored to achieve inflammation homing and lesion-specific therapeutic accumulation within vascular and tissue injury contexts [67]. Studies using neutrophil membrane-camouflaged nanoparticles demonstrate enhanced functional delivery and accelerated recovery in inflammatory disease and tissue repair models [14]. Engineering strategies applying neutrophil membrane-coated platforms have also enabled precise anti-tumor drug delivery and immune modulation within the tumor microenvironment [68].

In contrast, T cell- and B cell-derived membranes are less commonly utilized in therapeutic nanocarriers because their primary function is antigen-specific adaptive immunity rather than inflammation homing or tissue repair mechanisms [6,17]. Moreover, T cell or B cell membranes require complex antigen presentation states and may carry high immunogenic risk due to extensive TCR/BCR variability, limiting their practicality for broad or off-the-shelf therapeutic design [18]. Therefore, this review focuses on innate immune-derived leukocyte membranes that actively participate in inflammation resolution and tissue regeneration, rather than adaptive lymphocyte-based systems. Leukocyte-derived membranes are categorized based on hematopoietic origin and immunological function, rather than strict hematological definitions based on peripheral blood cell composition.

#### 3.3.1. Monocyte/Macrophage Membrane

While mature macrophages are not present as circulating leukocytes in peripheral blood, they originate from monocytes and share a common hematopoietic lineage [69]. Accordingly, macrophage membranes used in nanocarrier fabrication are typically derived from differentiated monocyte/macrophage cell lines or tissue-resident mononuclear cells rather than directly isolated from peripheral blood.

Macrophage membrane-coated nanoparticles exploit the innate immune surveillance and inflammation-homing capacity of macrophages toward inflammatory and pathological sites. The macrophage plasma membrane carries a variety of surface receptors such as integrins, scavenger receptors, and pattern-recognition molecules including CD14 and CD206, which enable specific adhesion to activated endothelium and recognition of pathogen- or damage-associated molecular patterns [15,70]. When transferred onto nanoparticle cores, these membrane components endow the nanocarrier with immune evasion and selective accumulation within inflamed tissues. Moreover, macrophage membranes can sequester inflammatory cytokines and chemokines, thereby attenuating excessive immune activation and contributing to a local anti-inflammatory microenvironment [70]. Several preclinical studies have demonstrated that macrophage membrane-coated nanoparticles improve drug delivery efficacy in inflammatory and infectious disease models. In ischemic or atherosclerotic tissues, macrophage membranes guide nanoparticles across endothelial barriers and reduce oxidative stress and cytokine release by acting as decoys for proinflammatory mediators [66]. Recent work using macrophage membrane-coated nanoparticles to deliver the neddylation inhibitor MLN4924 in diabetic wound models demonstrated dual immunoregulatory and regenerative effects, including suppression of NF-κB signaling, promotion of macrophage M2 polarization, enhanced angiogenesis, and increased collagen deposition, ultimately accelerating wound closure and tissue repair [71]. Collectively, these findings highlight macrophage membrane-coated nanoparticles as versatile biomimetic platforms capable of simultaneously modulating immune responses and supporting tissue regeneration.

#### 3.3.2. Neutrophil Membrane

While macrophage membranes offer broad interaction with inflammatory microenvironments and cytokine-buffering capacity, neutrophil membranes provide rapid recruitment and deep penetration into highly inflamed tissues, enabling more precise lesion targeting. Neutrophil membrane-coated nanoparticles inherit the unique migratory and inflammation-homing properties of neutrophils, the first responders in acute immune reactions [70,72,73]. Neutrophil membranes display chemokine receptors (CXCR1, CXCR2) and adhesion molecules (L-selectin, β2-integrins) that mediate firm binding to activated endothelial cells and directed migration toward chemotactic gradients [74,75]. By coating nanoparticle cores with neutrophil membranes, the resulting neutrophil membrane-coated nanoparticles can traverse vascular barriers and accumulate at sites of acute inflammation or tumor lesions where conventional synthetic carriers cannot easily reach [16,76,77]. In addition to passive targeting, neutrophil membranes act as decoys that neutralize pro-inflammatory cytokines and chemokines, thereby dampening the recruitment of excessive immune cells and reducing tissue damage [72,73].

Therapeutically, neutrophil membrane-coated nanoparticles have been applied in several inflammation-related diseases. For example, in a murine model of acute lung injury induced by LPS, neutrophil-membrane-coated PLGA nanoparticles loaded with TLR4 siRNA (Neutrophil-NP-TLR4) preferentially accumulated in inflamed lung tissue, reduced expression of TNF-α and IL-1β, suppressed downstream TLR4/TRAF6/NF-κB signaling and elevated expression of AQP1/AQP5, thereby attenuating pulmonary inflammation without overt toxicity [76]. In a collagen-induced arthritis model, neutrophil membrane-coated nanoparticles inhibited synovial inflammation, neutralized pro-inflammatory cytokines, reduced joint swelling and prevented cartilage/bone erosion by virtue of deep penetration into the synovium and targeting of activated endothelium. Moreover, neutrophil membrane-coated nanoparticles are increasingly explored in oncology. Owing to the intrinsic tumor-infiltrating nature of neutrophils, neutrophil-derived or membrane-coated nanocarriers can deliver chemotherapeutics or immunomodulators into the tumor microenvironment with high precision and offer the potential to combine drug delivery with immune modulation. These attributes collectively make neutrophil membrane-coated nanoparticles a powerful platform for targeted drug delivery and inflammation resolution [16,78].

#### 3.3.3. Dendritic Cell Membrane

Dendritic cell membranes introduce antigen-presenting and immunoregulatory capabilities that bridge innate and adaptive immunity, thereby expanding opportunities for precision immune modulation in tissue repair and disease control [79]. Notably, current reports predominantly focus on their roles in cancer immunotherapy, whereas direct applications in anti-inflammatory or tissue-regenerative settings remain limited. Dendritic cell membranes confer antigen-presenting and immunomodulatory properties to nanoparticles by transferring essential surface molecules such as MHC I/II, CD80, and CD86 [80]. These ligands allow dendritic cell membrane-coated nanoparticles to interact directly with T-cells and other immune subsets, functioning as biomimetic antigen-presenting nanocarriers. Recent studies have demonstrated the therapeutic potential of dendritic cell membrane-coated nanoparticles in cancer immunotherapy and immune regulation. When loaded with tumor antigens, dendritic cell membrane-coated nanoparticles efficiently present antigenic peptides and co-stimulatory signals to cytotoxic T lymphocytes, enhancing anti-tumor immunity. For instance, in a glioma model, dendritic-cell-membrane-coated PLGA nanoparticles loaded with rapamycin (aDCM@PLGA/RAPA) achieved enhanced blood–brain barrier (BBB) penetration, provoked robust CD8^+^ T-cell responses, and significantly prolonged survival [81]. Other reports have combined dendritic cell membrane-coated nanoparticles with chemo- or photodynamic therapies to synergistically induce immunogenic cell death and systemic immune memory [79,82].

Depending on the maturation state of the originating dendritic cells, the resulting membranes can either activate adaptive immunity or induce immune tolerance, offering a tunable interface for both immunostimulation and immunosuppression [83]. Beyond oncology, tolerogenic dendritic cell membrane-coated nanoparticles are beginning to attract interest as potential tools for autoimmune and allergic diseases, as well as transplant immunomodulation. Although no fully validated or published therapeutic systems have yet been reported, their conceptual ability to direct immune homeostasis through controlled antigen presentation has prompted early-stage exploration. Thus, dendritic cell membrane-coated nanoparticles represent a promising, yet still largely investigational, class of biomimetic nanoplatforms that bridge nanotechnology and adaptive immunology.

### 3.4. Hybrid and Engineered Membranes

Hybrid and engineered membrane systems strategically integrate the complementary biological functions of distinct blood-cell sources to generate multifunctional nanocarrier interfaces. Among them, the RBC–platelet hybrid membrane is the most extensively characterized, combining the prolonged circulation and immune-stealth properties of RBCs with the adhesive and pro-regenerative attributes of platelet-derived surfaces [84,85]. Such hybrid coatings have demonstrated notable efficacy in cardiovascular repair, wherein RBC–platelet hybrid membrane-coated nanoparticles preferentially localize to ischemic myocardium, suppress fibroblast activation, and mitigate adverse post-infarction remodeling. In vascular injury models, platelet-associated integrins and selectins promote adhesion to damaged endothelium, whereas RBC-derived CD47 signaling attenuates phagocytic clearance, collectively facilitating re-endothelialization and tissue restoration [10,57]. RBC–leukocyte hybrid membranes have, to date, been validated primarily in diagnostic settings. These systems have been applied to achieve high-purity enrichment of fetal nucleated RBCs for noninvasive prenatal testing [86]. No therapeutic or immuno-regenerative RBC–leukocyte hybrid nanoparticle has yet demonstrated in vivo efficacy. Nonetheless, their theoretical potential is compelling. RBC membranes provide immune-evasive characteristics and reduced protein fouling, while leukocyte membranes present chemokine receptors such as CXCR2 and CD11b that mediate active recruitment to inflamed microenvironments [13,74,75]. Additionally, leukocyte-derived anti-inflammatory programs including M2-like macrophage polarization and IL-10/TGF-β–driven suppression of TNF-α signaling are well documented in leukocyte-based membrane systems, suggesting future applicability in immuno-regenerative nanomedicine [70,76]. Platelet–leukocyte hybrid constructs, although primarily utilized in macroscale biomaterials rather than nanoparticle systems, provide further evidence of synergistic regenerative activity. Platelet- and leukocyte-rich fibrin matrices have been shown to enhance osteogenesis, regulate inflammatory responses, and promote structural healing in bone and dental applications [63]. Platelet-derived components supply adhesive interactions and growth factors such as PDGF, TGF-β, and VEGF [57], whereas leukocyte-related receptors and cytokines contribute to immune modulation and cellular recruitment within healing tissues [13,66]. Recent reviews likewise emphasize the complementary roles of platelet integrins/selectins and leukocyte chemokine receptors in governing adhesion and inflammation-homing functions [57,87]. Macrophage-based hybrid membranes have emerged as an additional class of immune-regenerative interfaces. In cardiovascular and ischemic disorders, macrophage membranes confer intrinsic capacity to buffer pro-inflammatory cytokines and tolerate oxidative stress, while incorporation of RBC or platelet layers improves systemic stability and vascular compatibility [70]. Collectively, these engineered hybrids illustrate the feasibility of designing blood cell-derived membranes as active immunomodulatory platforms for tissue repair [29,41].

To enable systematic comparison, Table 1 compiles representative nanocarriers derived from RBC, platelet, leukocyte (macrophage, neutrophil, dendritic cell), and hybrid membrane systems. Information on nanoparticle type, membrane-coating strategy, therapeutic payload, and disease model is provided to highlight structure–function relationships and translational opportunities in immune-regenerative nanomedicine.

## 4. Translational Challenges

The clinical translation of blood cell membrane-coated nanoparticles has gained attention for integrating immune evasion, prolonged circulation, and tissue-specific targeting in a single platform. However, unlike synthetic nanocarriers, cell membrane-coated nanoparticles rely on biologically derived materials that introduce variability, complexity, and regulatory hurdles. Key challenges include scalable manufacturing, pharmacological consistency, and safety validation.

### 4.1. Manufacturing and Scalability, and Quality Control

Large-scale production remains a major barrier. Membrane composition varies by donor age, health, and physiology, affecting protein and lipid profiles and resulting in inconsistent pharmacokinetics and efficacy [30]. RBCs and platelets are easier to obtain, whereas immune cell membranes from neutrophils, macrophages, or NK cells are limited by short lifespan and culture difficulty, complicating reproducibility [90]. To standardize sources, continuous bioreactors and immortalized cell lines are being explored. Hematopoietic stem cell expansion can generate RBCs with uniform membrane composition, while stable macrophage- or NK-like lines may yield consistent immune membranes [91]. Downstream steps must comply with GMP, ensuring sterile handling, closed-loop purification, and monitoring of protein integrity and endotoxin content. Microfluidic coating systems can improve scalability by enabling continuous, controlled membrane fusion and reducing batch variation [92,93]; however, industrial adaptation still requires validation to confirm reproducibility at production scale.

Beyond sourcing, quality control of membrane integrity and functionality represents a critical and still evolving challenge. Native blood cell membranes exhibit pronounced lipid asymmetry, defined protein orientation, and higher-order structures such as lipid rafts, all of which contribute to membrane-associated biological functions [94,95]. During membrane isolation, ghost formation, and nanoparticle assembly, these structural features may be partially altered, redistributed, or lost. As a result, direct molecular-level validation of membrane asymmetry, protein orientation, phosphorylation status, and raft preservation remains technically challenging [1]. Current quality control strategies therefore rely largely on indirect functional assays such as immune evasion, targeting efficiency, cytokine modulation, or circulation half-life, rather than comprehensive structural verification of membrane organization. While these functional readouts suggest partial retention of membrane activity, standardized analytical methods to quantitatively assess membrane orientation and higher-order organization are still limited and represent an important gap for industrial translation [96,97]. Integration of such systems with robust quality control pipelines will be essential to ensure batch-to-batch consistency and regulatory acceptance at manufacturing scale.

### 4.2. Pharmacology and Biodistribution

Pharmacological performance depends on both the membrane source and nanoparticle core. RBC membranes extend circulation via CD47, platelet membranes enhance vascular adhesion, and immune membranes enable homing to inflamed tissues; however, these effects vary with cytokine profiles, disease stage, and immune context, complicating study comparisons [30]. Circulation half-life and biodistribution profiles vary substantially depending on the membrane source. RBC membranes, reflecting the long physiological lifespan of erythrocytes in circulation, generally confer the most pronounced extension of systemic circulation through mechanisms such as CD47-mediated inhibition of phagocytic clearance [10,12]. In contrast, platelet membrane-coated nanoparticles typically exhibit relatively shorter circulation profiles but demonstrate superior vascular adhesion and lesion-specific accumulation owing to integrin- and selectin-mediated interactions with injured endothelium [58,61,62]. These differences highlight a fundamental design trade-off among blood cell membrane coatings, wherein prolonged circulation and active lesion targeting represent complementary rather than competing strategies. Accordingly, the choice of membrane source should be guided by therapeutic objectives, such as systemic exposure versus localized delivery, rather than by circulation time alone [84,85].

Hybrid membranes combine complementary properties such as RBC–platelet coatings merging immune evasion with vascular targeting, to improve biodistribution and efficacy [92,93,98]. Yet, increased compositional complexity challenges characterization and reproducibility [99]. Each source contributes distinct biomolecules, hindering pharmacokinetic prediction [100]. Thus, standardized assays for circulation, organ accumulation, and drug release kinetics are essential [101]. Reliable in vivo models must also account for interspecies differences in immune interactions, as murine data often diverge from human outcomes [102]. Advanced imaging tools, including PET, MRI, and fluorescence tomography, enable real-time tracking of nanoparticle fate and can bridge preclinical and clinical findings [103].

### 4.3. Safety, Immunogenicity, and Regulatory Considerations

Safety and immunocompatibility are critical for all biologically sourced therapeutics. Cell membrane-coated nanoparticles inherit both benefits and variability from their cellular origins; even minor compositional shifts can affect immune recognition and cytokine response. In the case of RBC membrane-based nanocarriers, antigen compatibility represents an additional immunological consideration. RBC membranes inherently express blood group antigens, including ABO and Rh antigens, which may trigger immune recognition or hemolytic reactions when non-matched or allogeneic sources are used. While most preclinical studies rely on syngeneic or species-matched models, these antigen-related risks may become more pronounced during clinical translation, particularly for off-the-shelf or allogeneic products. Potential strategies to mitigate blood group incompatibility include the use of universal donor-type membranes, enzymatic removal or masking of surface antigens, membrane engineering to reduce antigen exposure, or the development of autologous membrane-derived systems. Addressing blood group antigen compatibility will therefore be essential for ensuring the safety and translational feasibility of RBC membrane-coated nanoparticles [104,105]. Autologous membranes may offer excellent biocompatibility but are impractical for large-scale or urgent use [104]. Allogeneic or xenogeneic membranes are easier to source but raise antigen mismatch and residual donor protein concerns [105]. Chemical modification or hybridization may alter natural protein orientation or expose new epitopes, while oxidation or denaturation during storage can reduce tolerance. Quality criteria must ensure proper protein orientation, activity retention, and removal of nucleic acids and cytosolic residues to prevent inflammation or genetic transfer. Endotoxin levels should remain below 0.5 EU mL^−1^, verified via Limulus Amebocyte Lysate or recombinant Factor C assays [106]. Storage stability, sterility, and batch consistency should be confirmed using proteomic and physicochemical analyses. Leukocyte-derived membranes, being contamination-sensitive, require aseptic handling and routine tests for mycoplasma, sterility, and residual DNA. Regulatory agencies such as the FDA and EMA are developing frameworks bridging biologics and nanomedicine. Manufacturers must comply with GMP, validated potency and safety testing, and immunogenicity assessment [93]. Early engagement with regulators and robust quality systems will facilitate alignment and accelerate approval. Ultimately, advancing blood cell membrane-coated nanoparticles toward clinical use depends on demonstrating that biologically derived nanomaterials can meet the same rigor, reproducibility, and safety as conventional pharmaceuticals.

## 5. Future Perspectives

The development of blood cell membrane-coated nanoparticles is advancing rapidly through the convergence of biomaterials, immunology, and nanotechnology. While preclinical data demonstrate therapeutic potential, clinical translation requires enhanced reproducibility, scalability, and long-term safety. Among blood cell-derived platforms, platelet membrane-coated nanoparticles have progressed furthest toward clinical evaluation. Notably, a platelet membrane-based nanotherapeutic (CE120), developed by Cello Therapeutics, has been reported to receive investigational new drug (IND) clearance from the U.S. Food and Drug Administration (FDA) for oncology indications, representing an early example of a membrane-coated nanoparticle system entering a formal regulatory pathway [105]. Although this program is not specifically directed toward regenerative medicine, it establishes important regulatory, manufacturing, and quality-control precedents for blood cell membrane-based nanomedicines and therefore serves as a realistic translational benchmark for broader therapeutic applications.

Building on these early regulatory advances, further clinical translation will likely depend on rational membrane engineering strategies that extend beyond single-cell-type functionality. The rational design of hybrid and genetically engineered membranes represents a promising strategy to integrate complementary biological functions from different blood cell sources. For example, RBC membranes provide immune evasion, platelet membranes support vascular adhesion, and leukocyte membranes enable homing to inflammatory sites; the combination of these features through hybrid membrane architectures can yield multifunctional nanoparticles with improved biodistribution and therapeutic efficacy [29,107]. Cellular genetic engineering further expands possibilities. CRISPR-based modification can introduce therapeutic ligands or targeting peptides, such as tumor-recognizing receptors similar to CAR-T designs [108,109]. In parallel, integration with advanced biomaterials such as hydrogels or tissue scaffolds enables localized and sustained delivery, supporting spatially controlled immune modulation and tissue regeneration, as seen in platelet- or macrophage-derived systems enhancing wound healing and immune modulation [110].

Importantly, translational success will depend on regulatory alignment. Agencies such as the FDA and European Medicines Agency (EMA) are establishing quality standards for biologically derived nanomedicines, emphasizing GMP compliance, potency validation, and reproducibility [5,104]. Within this evolving regulatory landscape, blood cell membrane-coated nanoparticles are likely to follow two complementary developmental paths: personalized autologous formulations for oncology and immune-related disorders, and allogeneic or universal membrane platforms designed for large-scale treatment of cardiovascular, neural, and inflammatory diseases [104,111,112]. Accordingly, membrane-coated nanoparticles should be regarded not as replacements for cell-based therapies, but as complementary platforms that balance biological functionality with pharmaceutical practicality. Interdisciplinary collaboration, together with computational and AI-assisted predictive tools, will be essential to accelerate optimization and standardization. From a formulation perspective, blood cell membrane-coated nanoparticles are conceptually distinct from cell-based therapeutic products such as packed RBC transfusions, platelet-rich plasma, or CAR-T cell therapies. Unlike living cell formulations, membrane-coated nanoparticles are non-cellular systems that decouple selected membrane functions from cellular viability, enabling improved physicochemical stability, simplified storage requirements, and potentially greater manufacturability. Nevertheless, blood cell membranes may contain blood group antigens and immunologically relevant surface components; therefore, particularly in the development of allogeneic or off-the-shelf formulations, careful evaluation and optimization of immune compatibility will be an important consideration during translational development. If these challenges are met, blood cell membrane-coated nanoparticles could transition from experimental innovation to clinically established therapeutics, reshaping precision and regenerative medicine.

## 6. Conclusions

Blood cell membrane-coated nanoparticles represent a new paradigm in therapeutic delivery, merging the biological sophistication of living cells with the controllable design of synthetic nanomaterials. By retaining native surface proteins and signaling pathways, these biomimetic carriers achieve immune evasion, prolonged circulation, and targeted tissue interaction, which are the features long pursued by conventional nanocarriers. Utilizing RBC, platelet, and immune cell membranes enables broad applications in inflammation control, vascular repair, cancer therapy, and regenerative medicine. Yet translation from laboratory to clinic remains challenging due to variability in biological sources, limited large-scale membrane preparation, and the absence of unified quality and safety standards. Progress will depend on standardized protocols for membrane isolation, coating, and characterization, and on GMP-compliant production. Defining critical quality attributes, membrane integrity, protein orientation, and acceptable endotoxin levels will be key to ensuring consistent performance and safety. The future of this field will likely follow two paths: personalized autologous therapies and universal off-the-shelf platforms. Advances in genetic and material engineering promise programmable targeting, immune modulation, and regenerative functions, while computational modeling, microfluidic fabrication, and advanced analytics will support scalable, reproducible manufacturing. Ultimately, collaboration among materials scientists, immunologists, and clinicians will be essential as evolving technologies and regulations move these biomimetic systems toward clinical reality in precision and regenerative medicine.

## Figures and Tables

**Table 1:** Blood cell membrane–coated nanoparticles (NP) and their therapeutic applications.

Membrane Type	Membrane Subtype	Core NP	Fusion/Coating Method	Drug (Specific Payload)	Target Organ/Disease	Ref.
RBC Membrane	-	Polymer-based nanoframework	Extrusion	MSC-derived growth factors	Liver/Acute liver failure	[42]
		PLGA		MSC-secretomes		[43]
		Dextran polymer NP	Sonication	NR2B9C	Brain/Ischemic stroke	[44]
Platelet Membrane	-	PLGA	Sonication	Fat extract	Brain/Ischemic stroke	[58]
			Extrusion + Sonication	bFGF + VEGFA plasmid DNA	Skin/Deep burn wound	[59]
		Polyethyleneimine	Extrusion	TGF-β1 siRNA	Kidney/Renal inflammation	[60]
WBC	Macrophage membrane	PLGA	Extrusion + Sonication	MLN4924	Skin/Diabetic wound	[71]
	Neutrophil membrane	PLGA	Sonication	IL-5	Heart/Ischemic myocardium	[72]
			Extrusion	Simvastatin	Blood vessel/Atherosclerosis	[77]
	Dendritic cell membrane	Polyethyleneimine	Extrusion	Ovalbumin mRNA	Lymphoid tissue/Melanoma	[80]
Hybrid Membranes	RBC/ Platelet membrane	PLGA	Sonication	None	Blood vessel/Atherosclerosis	[84]
				BET protein inhibitor JQ1	Heart/Ischemic myocardium	[85]
	RBC/Neutrophil membrane	Hollow CuS NP	Sonication	Dexamethasone sodium phosphate	Joint/Osteoarthritis	[88]
	Platelet/Macrophage membrane	Benzyl-modified helical polypeptide	Sonication	Sav1 siRNA	Heart/Ischemic myocardium	[89]

## Data Availability

No new data were created or analyzed in this study.
